# European Robotic Surgery Consensus (ERSC): Protocol for the development of a consensus in robotic training for gastrointestinal surgery trainees

**DOI:** 10.1371/journal.pone.0302648

**Published:** 2024-05-31

**Authors:** Michael G. Fadel, Josephine Walshaw, Francesca Pecchini, Muhammed Elhadi, Marina Yiasemidou, Matthew Boal, Francesco Maria Carrano, Lisa H. Massey, Stavros A. Antoniou, Felix Nickel, Silvana Perretta, Hans F. Fuchs, George B. Hanna, Nader K. Francis, Christos Kontovounisios, On behalf of the European Robotic Surgery Consensus (ERSC) study group

**Affiliations:** 1 Department of Surgery and Cancer, Imperial College London, London, United Kingdom; 2 Leeds Institute of Medical Research, St James’s University Hospital, University of Leeds, Leeds, United Kingdom; 3 Division of General Surgery, Emergency and New Technologies, Baggiovara General Hospital, Modena, Italy; 4 Tripoli University Hospital, Tripoli, Libya; 5 The Royal London Hospital, Barts Health NHS Trust, London, United Kingdom; 6 The Griffin Institute, Northwick Park and St Mark’s Hospital, London, United Kingdom; 7 Department of Medical and Surgical Sciences and Translational Medicine, Faculty of Medicine and Psychology, St Andrea Hospital, Sapienza University, Rome, Italy; 8 Department of Colorectal Surgery, Nottingham University Hospitals NHS Trust, Nottingham, United Kingdom; 9 Department of Surgery, Papageorgiou General Hospital, Thessaloniki, Greece; 10 Department of General, Visceral and Thoracic Surgery, University Medical Center Hamburg-Eppendorf, Hamburg, Germany; 11 IRCAD, Research Institute Against Digestive Cancer, Strasbourg, France; 12 NHC University Hospital, Strasbourg, France; 13 Department of General, Visceral, Cancer and Transplantation Surgery, University Hospital Cologne, Cologne, Germany; 14 Department of Colorectal Surgery, Chelsea and Westminster Hospital NHS Foundation Trust, London, United Kingdom; 15 Department of Colorectal Surgery, Royal Marsden NHS Foundation Trust, London, United Kingdom; 16 2nd Department of Surgery, Evangelismos Hospital, Athens, Greece; Acibadem Maslak Hospital: Acibadem Maslak Hastanesi, TURKEY

## Abstract

**Background:**

The rapid adoption of robotic surgical systems across Europe has led to a critical gap in training and credentialing for gastrointestinal (GI) surgeons. Currently, there is no existing standardised curriculum to guide robotic training, assessment and certification for GI trainees. This manuscript describes the protocol to achieve a pan-European consensus on the essential components of a comprehensive training programme for GI robotic surgery through a five-stage process.

**Methods and analysis:**

In Stage 1, a Steering Committee, consisting of international experts, trainees and educationalists, has been established to lead and coordinate the consensus development process. In Stage 2, a systematic review of existing multi-specialty robotic training curricula will be performed to inform the formulation of key position statements. In Stage 3, a comprehensive survey will be disseminated across Europe to capture the current state of robotic training and identify potential challenges and opportunities for improvement. In Stage 4, an international panel of GI surgeons, trainees, and robotic theatre staff will participate in a three-round Delphi process, seeking ≥ 70% agreement on crucial aspects of the training curriculum. Industry and patient representatives will be involved as external advisors throughout this process. In Stage 5, the robotic training curriculum for GI trainees will be finalised in a dedicated consensus meeting, culminating in the production of an Explanation and Elaboration (E&E) document.

**Registration details:**

The study protocol has been registered on the Open Science Framework (https://osf.io/br87d/).

## Introduction

In the last two decades, there has been a rapid growth in the adoption of robotic systems in centres across Europe [1, 2]. Robotic surgery has several technical advantages over conventional techniques for certain procedures which may potentially improve surgical quality and oncological outcomes [3–5]. These advantages stem from features such as three-dimensional visualisation, articulating instruments, and enhanced access to complex or narrow anatomical areas [6, 7]. Additionally, robotic systems enable direct camera control, tremor reduction, and decrease surgeon muscle strain and fatigue [8, 9]. However, longer operative times and higher costs are associated with robotic surgery when compared to laparoscopy [10].

Robotic surgery has a set of skills that are distinct to those required for laparoscopic and open surgery. The development of these skills requires training through a dedicated and structured programme, covering essential knowledge, safety principles, and the necessary technical skills required to achieve optimal surgical outcomes. Currently, there is significant variability in robotic console training methods and tools being adopted, including dry skills laboratory (synthetic models on robotic consoles), wet skills laboratory (porcine or cadaveric models) and virtual reality simulation [11, 12]. Patient side training is required which involves patient positioning, establishing pneumoperitoneum, procedure specific port placement and robot docking. The development of non-technical skills is also an essential part of training and should occur in parallel.

In 2014, the European Association of Endoscopic Surgery (EAES) society created a consensus statement on the use of robotics in general surgery [13]. This primarily focused on robotic set-up, dexterity, ergonomics, and clinical applications such as oesophago-gastric, cholecystectomy, hepatobiliary, colorectal and bariatric surgery. At the time, the education, training, assessment, and certification process for robotic surgery had not been thoroughly evaluated due to its relative infancy and the acceptance of robotics within the surgical community was uncertain. Thus far, training in robotics in general surgery has heavily relied on industry-led courses and independent fellowships without a formal proficiency-based curriculum and validated accreditation process [14–16].

Some groups have developed training programmes for highly sophisticated surgical approaches [17–22], however, there is an undoubted need for a standardised basic robotic training curriculum. Currently, there is no consensus among societies and surgical specialty boards regarding training and certification for gastrointestinal (GI) trainees in robotic surgery. This study describes a protocol to develop a European consensus on robotic training, assessment and certification for GI robotic trainees. A standardised curriculum that enables GI trainees to attain certification in robotics will ultimately improve robotic training and enhance clinical and patient outcomes.

## Materials and methods

**Fig 1:**
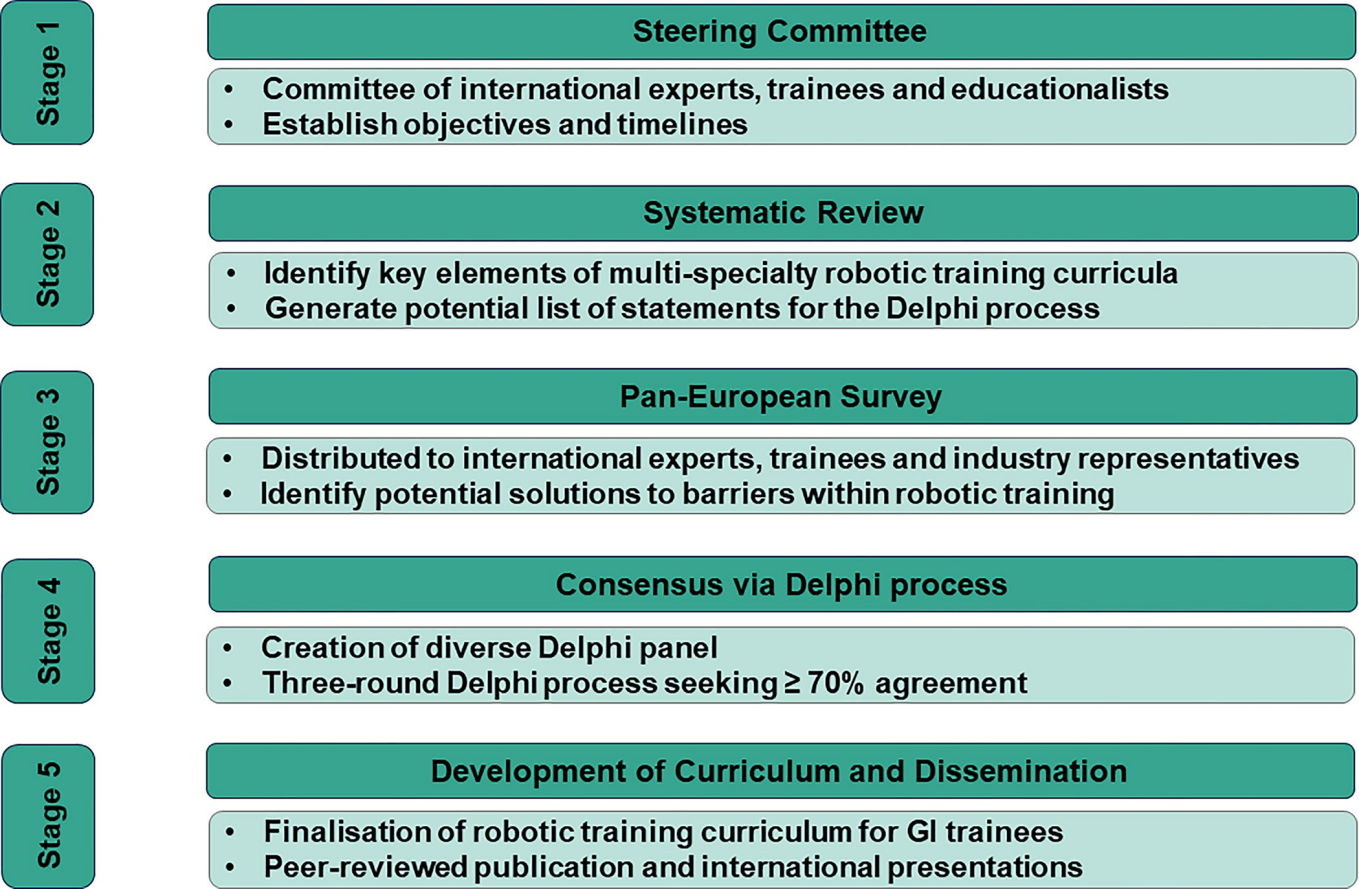
The five key stages to reach a European consensus in robotic training for GI trainees. GI, gastrointestinal.

The European consensus in robotic training for GI trainees will be led by EAES and developed through five key stages (Fig 1) described below. The study protocol has been registered on the Open Science Framework [23] and has been reported in line with the proposed steps of the Accurate Consensus Reporting Document (ACCORD) guidelines [24] and applicable items of Appraisal of Guidelines, Research and Evaluation (AGREE) II [25] to integrate opinions from stakeholders and experts in the field.

### Stage 1: Establishment of steering committee

We have formed a European Robotic Surgery Consensus (ERSC) Steering Committee [MGF, JW, FP, ME, MY, MB, FMC, LHM, SAA, FN, SP, HFF, GBH, NKF, CK], which will lead in coordinating the consensus development process. It is an international multidisciplinary diverse group comprising of 15 members, including upper and lower GI surgical experts and trainees, evidence synthesis and educational experts and methodologists, chairs and members from the EAES committees, including the Guidelines, Technology and Research Committees, Artificial Intelligence (AI) and Robotics Subcommittees and the Executive Board. The Steering Committee are responsible for establishing the objectives and timelines for the study, with the study expected to be completed by April 2025. The conduction of a relevant systematic review, pan-European survey and a Delphi consensus process will be supervised and managed by the committee, who will also be responsible for disseminating the consensus.

### Stage 2: Systematic review

A systematic review will be performed to identify and define the essential components of a robotic training curriculum across all surgical specialities, including current training methods and assessment tools. It will be reported according to the Preferred Reporting Items for Systematic Reviews and Meta-Analysis (PRISMA) 2020 guidelines [26] and developed in line with the Cochrane Handbook for Systematic Reviews of interventions [27]. The systematic review has been registered in the PROSPERO database (CRD42023418429).

#### Search strategy

A comprehensive literature search of MEDLINE, Embase, Emcare and CINAHL databases will be performed. Specific search equations will be formulated for each database using relevant Medical Subject Headings (MesH) terms including robotic surgery, robot-assisted surgery, training, simulation, syllabus, curriculum, fellowships, residency and education. We will retrieve articles published in the English language between 1^st^ January 2000 to 1^st^ February 2024, which report on robotic training and curricula in surgery. The reference lists of the selected studies will also be reviewed to identify any additional relevant studies.

#### Study selection and data extraction

All studies reporting on multi-speciality or specialty specific robotic training curricula (including single procedure curricula) in any surgical specialty will be eligible for inclusion. Training or assessment on cadaveric or porcine models will also be included. Study designs to be included are randomised controlled trials, prospective and retrospective cohort studies, expert opinion or Delphi methodology studies. The exclusion criteria will be the following: (i) laparoscopic or other non-robotic surgery, (ii) using robotics or simulation to train for non-robotic surgery, (iii) articles published in a non-English language and (iv) letters to the editor, case reports, reviews or conference abstracts.

Two authors will conduct the search and identification independently against the inclusion and exclusion criteria, arriving at a final list of articles. Any disagreement will be resolved by a third independent reviewer. The impact of curricula will be assessed for predictive validity within the clinical setting (intraoperative or patient outcome data). Data will be extracted according to Messick’s concept of validity [28] and Kirkpatrick’s model of curriculum evaluation [29]. For an assessment tool to be valid according to Messick’s concept of validity, the following five aspects will be considered: (i) test content (face and content validity), (ii) response process (analysis of raters), (iii) internal structure (reliability), (iv) relationship to other variables (predictive validity and learning curve) and (v) consequences (impact of assessment or curricula). Evaluation of each curriculum will be performed according to Kirkpatrick’s model: (i) reaction from participants (feedback), (ii) learning by participants measured objectively or subjectively, (iii) impact of training/curriculum on participant behaviour and (iv) long-term impact on participants’ learning/outcomes. The findings of the systematic review will be summarised in tables and will be used to generate the preliminary list of checklist items for the Delphi process.

#### Quality assessment of studies

Study quality will be assessed and quantified using the validated Medical Education Research Study Quality Instrument (MERSQI) [30]. The MERSQI tool consists of six scoring domains created to assess different aspects of medical education research. Domains include study design, sampling, type of data, validity of assessment instruments, data analysis and study outcomes. All studies will be rated independently by two authors, with any differences resolved by consensus. The overall certainty of the evidence will be assessed using the GRADE (Grading of Recommendations Assessment, Development and Evaluation) approach [31]. This assessment considers the risk of bias, inconsistency, indirectness, imprecision, and publication bias.

### Stage 3: Pan-European robotic training survey

The Steering Committee will design a pan-European survey which will be hosted by Qualtrics XM software [32], a secure web-based survey platform. The survey will be displayed in a branching format to account for each individual target group: (i) experts/independent practitioner with and without robotic access, (ii) trainees with and without robotic access, (iii) industry representatives. The survey will be specifically addressed to European countries and will be distributed to members of several European based surgical societies including, but not limited to, EAES, United European Gastroenterology (UEG), Royal College of Surgeons (RCS), Association of Laparoscopic Surgeons of Great Britain and Ireland (ALSGBI), Società Italiana di Chirurgia Endoscopica (SICE), German Society of Surgery Section of Computer and Telematic Assisted Surgery (CTAC), Professional Association of German Surgery (BDC), Belgian Robotic Surgery Working Group (RSWG), European Society of Coloproctology (ESCP) and the Research Institute against Digestive Cancer (IRCAD). The survey will also be distributed to other major international robotic societies such as Upper GI International Robotic Association (UGIRA). Two separate survey invitations will be sent via email, approximately four weeks apart, to maximise the response rate. Furthermore, the survey will be distributed through our official social media Twitter page (@ERSC_Study) and to robotic industry contacts directly.

The Steering Committee recognises the importance of capturing diverse perspectives to have a comprehensive understanding of the current state of robotic training and gain valuable insights into the barriers to training to identify potential solutions. The inclusion of independent robotic surgeons allows for the incorporation of their expertise and experiences in training aspiring robotic surgeons. Their perspectives can shed light on effective training methodologies and recommendations for improving the training process. Engaging trainees and surgeons with limited or no access to robotic systems is equally important. This will ensure the survey captures the viewpoints of individuals who are undergoing or seeking robotic training, but also those who face challenges in accessing the necessary resources. Understanding these perspectives can help inform initiatives to facilitate access to robotic training. Involving industry representatives is also crucial as they play a significant role in the development, distribution and assessment associated with robotic systems. Their input can provide awareness into policies and form potential collaborations with professional societies to enhance training opportunities and address access-related challenges. The responses of this survey will help inform the subsequent Delphi process in Stage 4.

### Stage 4: Delphi panel and consensus process

The Delphi methodology is a widely accepted technique for reaching a consensus among a panel of experts [33–36], enabling the collation of international opinions while being cost-effective and reducing the need to travel. The anonymous nature of the Delphi method also ensures that a single dominant group member does not inordinately influence the group’s outcome [37], with a minimum number of 20 participants previously been shown to provide reliable judgements [38, 39].

#### Delphi panel

**Fig 2:**
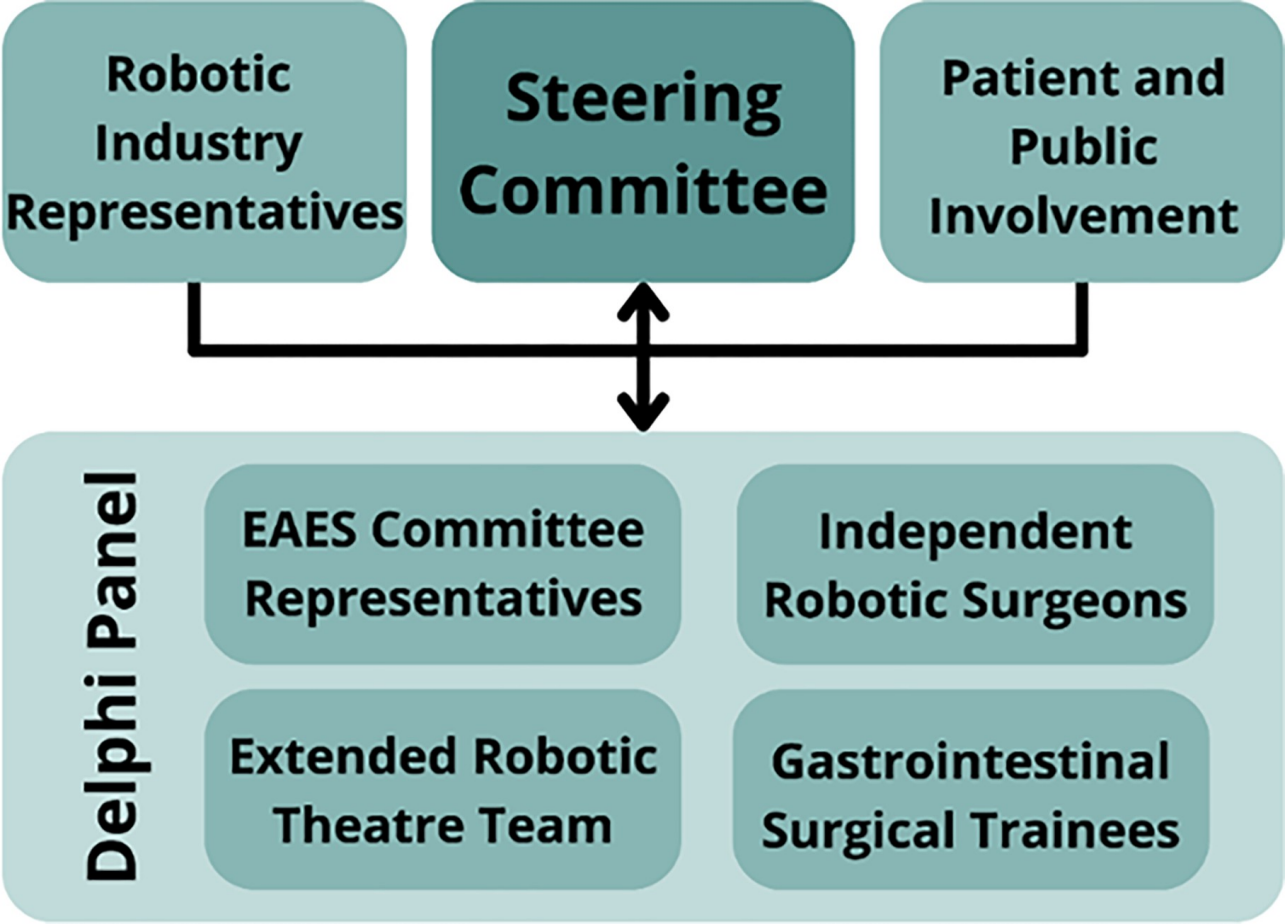
Key stakeholders involved in the development of a European consensus for robotic training in GI trainees. EAES, European Association of Endoscopic Surgery.

We will invite a diverse group of at least 40 participants with representation from at least 15 European countries. The panel will ensure there is a minimum of 8 representatives in each of the following categories: (i) expert/independent robotic upper GI surgeons; (ii) expert/independent robotic colorectal surgeons; (iii) GI surgical trainees including those with and without access to robotics; (iv) representatives from the extended robotic theatre team including anaesthetists, scrub nurses, and surgical first assistants (Fig 2). In addition, we will involve industry representatives as external advisors from at least three platform-producing companies such as Intuitive Surgical, Medtronic and CMR Surgical. There will also be patient advocates who will be actively incorporated in the consensus development process. They will help bring a valuable patient-centred perspective to the discussion regarding robotic training for GI surgical trainees. Their input will contribute to shaping a consensus that aligns with their expectations of how a robotic surgeon should be trained, as well as to promote patient-centred care and to enhance transparency.

During the Delphi process, there will be no direct or indirect conflicts of interest among the Steering Committee members, who will be asked to provide a written declaration prior to the commencement of the project. Stakeholders deemed to have potential direct or indirect conflicts, for example industry representatives, will participate as external advisors with no involvement in the decisions on the content of the statements. Conflicts of interests will be regularly mapped during the project until completion.

#### Delphi methodology

The Delphi process below will be employed to achieve consensus among the panel and will involve three rounds of voting, where the panel provide their feedback and opinions on the items. The questionnaires are iteratively administered via a web link by email. For each item, the Delphi panel will provide their responses using a 5-point Likert scale (e.g. 1 –Strongly Agree, 2 –Agree, 3 –Neutral, 4 –Disagree, 5 –Strongly Disagree). The consensus threshold will be defined as at least 70% of responding panellists voting ‘Strongly Agree’ or ‘Agree’. This level of agreement has been considered appropriate in previous Delphi studies [40–42]. Data on participant demographics will be collected including gender, year of birth, country residence, current job position and time (in years) working in the field of robotic surgery and we will ensure that all stakeholder categories are represented in each round.

Panellists will be encouraged to suggest additional statements or modifications to the statements in free text fields. Rounds 2 and 3 questions will be modified according to the comments and suggestions from previous rounds. Each round will be closed once 90% of the experts have responded. Results will be analysed, and those questions that failed to reach consensus will be evaluated, rephrased if necessary, and distributed to the panellists for rounds 2 and 3, in addition to any novel statements generated from the previous rounds. The Delphi process will close when all responses have reached consensus or agreed on non-consensus.

The chosen Delphi process will allow the Delphi panel to review and consider the opinions of others, stimulating further discussion and the opportunity to make additional suggestions or recommendations. A draft document will be prepared based on the consensus achieved through this method by the Steering Committee and it will incorporate the identified items and best practices for inclusion.

### Stage 5: Development of robotic curriculum for GI trainees

A final consensus in-person meeting will be conducted with the Delphi panel, where the draft document will be presented, and panellists will have the opportunity to provide their input, suggestions, and feedback. The purpose of this meeting is to ensure that the document represents a consensus among the panel and to address any remaining concerns or considerations. The opinions of all members of the panel will be integrated to arrive at a European consensus on a formal curriculum for robotic training for GI trainees and a separate Explanation and Elaboration document (E&E) will be produced by the Steering Committee. The consensus will ultimately consist of the essential curriculum content and training, minimum requirements and competency-based assessments including assessment tools to monitor performance.

### Ethics and dissemination strategy

Ethical approval is not required for the steps of this consensus study. Written informed consent and conflict of interest forms will be obtained from all participants in the Delphi process.

The dissemination of the European consensus in robotic training for GI trainees will be crucial for the implementation in practice. We will disseminate the consensus through official social media channels, such as Twitter (@ERSC_Study), the EAES website, and through industry providers and international societies. In addition, the consensus will be disseminated through peer-reviewed publication and international conferences.

## Discussion

Robotic training programmes have been previously proposed internationally, however, they have often lacked validation and objective metrics preventing full implementation of proficiency-based progression training [43–45]. For example, the Fundamentals of Robotic Surgery (FRS) was designed to develop a curriculum of basic skills applicable to all surgical specialties for training and assessing surgeons desiring to engage in robotic surgery [46–48]. However, completion of the FRS does not qualify the trainer to perform robotic surgery. It serves as a measurement tool to indicate proficiency in utilisation of the robot rather than competency in robotic surgery.

Previous studies have provided consensus recommendations for multispecialty robotic training [22, 49–51], however, currently there is no consensus for GI trainees in robotic surgery. The establishment of a consensus in this field is essential for standardising and enhancing the quality of training and addressing barriers faced by trainees, which should ultimately lead to an improvement in patient care and safety. A dedicated consensus for GI training will contribute to a steeper learning curve and help to address the specific advancements and updates in robotic surgery, to provide more targeted and relevant guidance for surgical trainees.

The European consensus will provide key clinical performance indicators or outcomes, such as the specific number of procedures required, optimal exposure time to simulators before real-time operating, training in docking, recommended fellowships, as well as setting the minimum hours of simulation for supervised and unsupervised practice. The consensus will also explore the value of diverse training modalities such as robotic basic simulation courses, cadaveric training, case observations with the multidisciplinary team in an expert centre, structured proctorship programmes, train the trainer courses, and modular training approaches.

In addition, we will gain further insight into assessment tools to achieve accreditation in robotic surgery. Assessment tools that will be evaluated include Global Assessment Scale (GAS), Global Evaluative Assessment of Robotic Skills (GEARS), procedure-specific assessment tools, patient outcomes or complications, and video assessment with or without the use of AI [52–56]. De Backer et al. [57] proposed an annotation guide that could provide the basis for AI-based quality assessment, including instrument detection, segmentation and pose estimation. As recently highlighted, manual and automated assessment tools for robotic surgery are not yet well validated and require further evaluation before use in the accreditation process [58]. Future research, beyond this consensus, should focus on validating existing assessment tools and developing and piloting a new AI-specific study quality assessment tool.

This consensus study will ensure that diverse perspectives are represented by involving members from various groups and societies across Europe, including expert/independent robotic surgeons, surgical trainees, the extended theatre team, industry and patient representatives. This inclusive approach enhances the relevance and applicability of the consensus recommendations, as it considers the insights and experiences of those directly involved in robotic training and the broader healthcare community. The collaboration with EAES will provide collective knowledge and experience through their Guidelines, Research, Technology, Education & Training Committees, as well as AI and Robotics Subcommittee representatives, further reinforcing the validity and credibility of the consensus provided.

Furthermore, the ERSC study group recognises the importance of addressing both technical and non-technical skills in robotic surgical education. By encompassing a holistic approach that goes beyond technical proficiency, the consensus aims to shape a comprehensive training framework. This consideration of non-technical skills for surgeons (NOTSS), such as communication, teamwork, and decision-making in the operating room, acknowledges the multifaceted nature of surgical practice [59, 60]. NOTSS will therefore be incorporated into the consensus recommendations with the aims to provide trainees with a well-rounded skill set, ultimately enhancing their ability to deliver safe surgical care.

### Limitations

The actual implementation and integration of the consensus into training programmes may face its own challenges. Several industry providers (e.g. Intuitive Surgical) have a pre-existing generic curriculum for robotic training which may provide potential barriers. Our study is also specific to the European region due to the major differences in robotic training and availability between parts of Europe, the United States of America and Asia.

## Conclusions

This study will provide the first European consensus and curriculum in robotic training for GI surgery trainees. This consensus will help shape the future of robotic surgical education, promote standardised training practices and improve patient safety. The collaborative and inclusive approach, incorporating diverse expertise and perspectives, ensures that the consensus will reflect the collective knowledge and experiences of the key stakeholder groups.

## Supporting information

S1 Checklist PRISMA-P (Preferred Reporting Items for Systematic review and Meta-Analysis Protocols) 2015 checklist: Recommended items to address in a systematic review protocol*. (DOC)
